# The kynurenine pathway of tryptophan metabolism: a neglected therapeutic target of COVID-19 pathophysiology and immunotherapy

**DOI:** 10.1042/BSR20230595

**Published:** 2023-08-07

**Authors:** Abdulla Abu-Bakr Badawy

**Affiliations:** Formerly School of Health Sciences, Cardiff Metropolitan University, Western Avenue, Cardiff CF5 2YB, Wales, U.K.

**Keywords:** 3-dioxygenase, 3-dioxygenase, Aryl hydrocarbon receptor, Indoleamine 2, Kynurenic acid, Poly (ADP-ribose) polymerase, Proinflammatory cytokines, Tryptophan 2

## Abstract

SARS-CoV-2 (COVID-19) exerts profound changes in the kynurenine (Kyn) pathway (KP) of tryptophan (Trp) metabolism that may underpin its pathophysiology. The KP is the main source of the vital cellular effector NAD^+^ and intermediate metabolites that modulate immune and neuronal functions. Trp metabolism is the top pathway influenced by COVID-19. Sixteen studies established virus-induced activation of the KP mediated mainly by induction of indoleamine 2,3-dioxygenase (IDO1) in most affected tissues and of IDO2 in lung by the increased release of proinflammatory cytokines but could additionally involve increased flux of plasma free Trp and induction of Trp 2,3-dioxygenase (TDO) by cortisol. The major Kyn metabolite targeted by COVID-19 is kynurenic acid (KA), the Kyn metabolite with the greatest affinity for the aryl hydrocarbon receptor (AhR), which is also activated by COVID-19. AhR activation initiates two important series of events: a vicious circle involving IDO1 induction, KA accumulation and further AhR activation, and activation of poly (ADP-ribose) polymerase (PARP) leading to NAD^+^ depletion and cell death. The virus further deprives the host of NAD^+^ by inhibiting its main biosynthetic pathway from quinolinic acid, while simultaneously acquiring NAD^+^ by promoting its synthesis from nicotinamide in the salvage pathway. Additionally, the protective effects of sirtuin 1 are minimised by the PARP activation. KP dysfunction may also underpin the mood and neurological disorders acutely and during ‘long COVID’. More studies of potential effects of vaccination therapy on the KP are required and exploration of therapeutic strategies involving modulation of the KP changes are proposed.

## Introduction

Whereas most recent studies of COVID-19 have focused on vaccine-related aspects, there is a significant minority of studies addressing changes in metabolism of the essential amino acid *L*-tryptophan (Trp) and modulation of immune responses by its kynurenine (Kyn) and other metabolites. It is therefore opportune to review a range of observations of changes reported in the Kyn pathway (KP) and its immune-active metabolites in the context of the immune status of COVID-19 infection that may play an important role in the pathophysiology, and point towards potential immunotherapy, of this pandemic. In this Review, a brief account of the KP and the immunomodulatory properties of its intermediates will be followed by a summary of initial observations before a discussion of subsequent supporting findings.

## The tryptophan-degradative pathways

**Figure 1:**
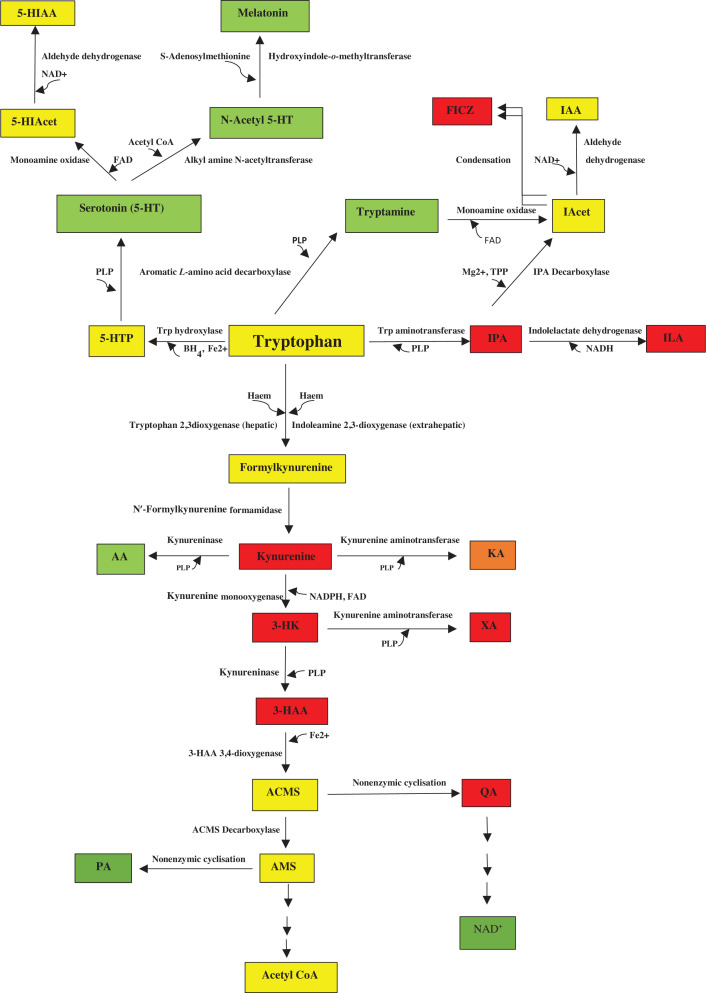
The tryptophan degradative pathways This Figure is reproduced from Figure 1 in [2] by Badawy, A.A.-B. (Tryptophan metabolism and disposition in cancer biology and immunotherapy. *Biosci. Rep*. **42**, 1–27. BSR20221682 https://doi.org/10.1042/BSR20221682). Abbreviations: 3-HAA, 3-hydroxyanthranilic acid; 5-HIAcet, 5-hydroxyindoleacetaldehyde; 5-HIAA, 5-hydroxyindoleacetic acid; 3-HK, 3-hydroxykynurenine; 5-HT, 5-hydroxytryptamine or serotonin; 5-HTP, 5-hydroxytryptophan; AA, anthranilic acid; Acet, acetaldehyde; ACMS, 2-amino-3-carboxymuconic acid-6-semialdehyde, also known as acroleyl aminofumarate; AMS, 2-aminomuconic acid-6-semialdehyde; IAcet, indole acetaldehyde; IAA, indol-3-ylacetic acid; ILA, indol-3-yllactic acid; IPA, indol-3-ylpyruvic acid; KA, kynurenic acid; PA, picolinic acid; QA, quinolinic acid; XA, xanthurenic acid. Colour code: red (proinflammatory); green (antiinflammatory); amber (dually acting); yellow (normal metabolite).

**Figure 2:**
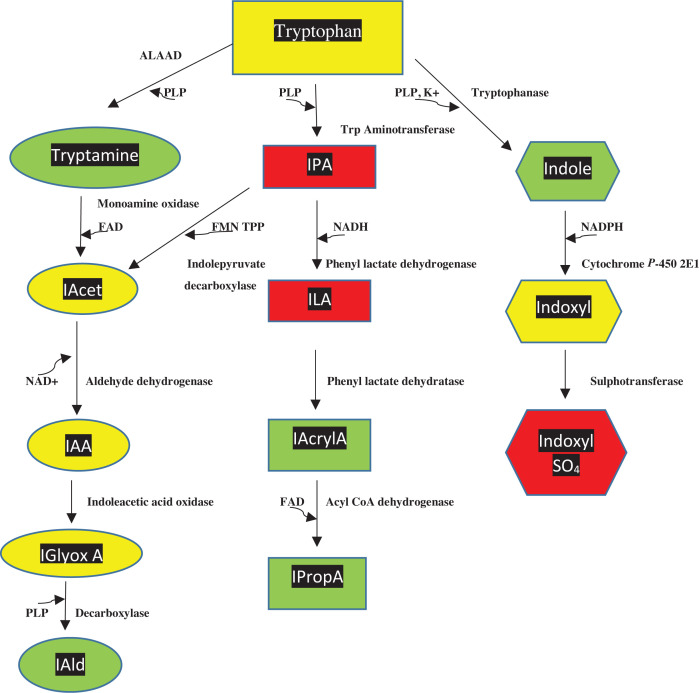
Tryptophan metabolism by gut microbiota This Figure is reproduced from Figure 2 in [2] by Badawy, A.A.-B. (Tryptophan metabolism and disposition in cancer biology and immunotherapy. *Biosci. Rep*. **42**, 1–27. BSR20221682 https://doi.org/10.1042/BSR20221682) Abbreviations: ALAAD, aromatic *L*-amino acid decarboxylase; FAD, flavin-adenine dinucleotide; FMN, flavin mono nucleotide; IAcet, indole acetaldehyde; IAA, indoleacetic acid; IAld, indole aldehyde; IAcrylA, indole acrylic acid; IGlyoxA, indole glyoxylic acid; ILA, indole lactic acid; IpropA, indole propionic acid; IPA, indole pyruvic acid; NAD(P)H, [reduced nicotinamide-adenine dinucleotide (phosphate)]; PLP, pyridoxal 5′-phosphate. Colour code: as in Figure 1.

Dietary tryptophan (Trp) is metabolised in mammals by four pathways (Figure 1) and by gut microbiota (Figure 2). Little Trp (1%, if at all) is utilised for protein synthesis, as, in a person in net nitrogen balance, the Trp released from protein breakdown is reutilised for protein synthesis [1]. The bulk of dietary Trp is, therefore, available for metabolism by the above four pathways (Figure 1), three of which contribute very little (1%) to Trp degradation, whereas the contribution of the fourth (the kynurenine pathway: KP) is ∼95% [2,3]. The three minor pathways are however of great functional significance. The hydroxylation pathway produces serotonin (5-hydroxytrytamine or 5-HT), which plays important roles in control of mood, emotions and other cerebral and peripheral functions. Serotonin can be further converted into melatonin in the pineal and periphery. The decarboxylation pathway converts Trp into tryptamine, which has the characteristics of a neurotransmitter and can be further metabolised to neuro- and immuno-active metabolites. The transamination pathway produces indol-3-ylpyruvic acid (IPA) that possesses a range of activities influencing brain and immune functions. Gut microbiota also metabolise Trp and some can also synthesise it. As outlined in Figure 2, microbiota process Trp in three main routes: decarboxylation to tryptamine, transamínation to IPA and hydrolysis to indole.

## The kynurenine pathway of tryptophan metabolism

### Outline of the pathway

The KP is responsible for 95% of dietary Trp degradation. The hepatic pathway accounts for 90% and the extrahepatic pathway contributes the remaining 5% [3]. The pathway is controlled mainly by the first enzyme, hepatic Trp 2,3-dioxygenase (TDO) and extrahepatic indoleamine 2,3-dioxygenase (IDO). IDO exists in two isoforms, IDO1 and IDO2, with the former being far more active than the latter. The basically low IDO1 activity acquires a greater quantitative significance following immune activation, wherein proinflammatory cytokines, notably interferon-γ (IFN-γ), are potent inducers. As depicted in Figure 1, Trp is the precursor of several KP metabolites. The Kyn formed is preferentially metabolised by oxidation by Kyn monooxygenase (KMO) to 3-hydroxykynurenine (3-HK) followed by hydrolysis of 3-HK to 3-hydroxyanthranilic acid (3-HAA) by the action of kynureninase (KYNU). Both Kyn and 3-HK can also be transaminated by Kyn aminotransferase (KAT) to kynurenic acid (KA) and xanthurenic acid (XA) respectively. Oxidation by KMO and hydrolysis by KYNU are favoured over transamination by KAT because of the greater affinity of the former 2 enzymes towards their respective substrates, compared with those of KAT for its two substrates [3]. Thus, meaningful increases in KA and XA levels require significant increases in levels of their precursors. KYNU can also convert Kyn to anthranilic acid (AA), though in humans, the main KYNU reaction is that of 3-HK to 3-HAA, because of the greater affinity of the enzyme for its 3-HK (*K*_m_ = 77 µM) than its Kyn (*K*_m_ = 1 mM) substrate [3], with an activity ratio of 15:1 [4]. This is further supported by the findings in 114 healthy volunteers of fasting plasma ratios of [3-HAA]/[3-HK] versus [AA/][Kyn] of 28:1, with women having a ratio double that of men (40:1 vs. 20:1) [5]. In this latter study [5], Caucasian females exhibited a significantly higher [3-HAA]/[AA] ratio.

3-HAA undergoes oxidation by 3-HAA 3,4-dioxygenase (3-HAAO) to the unstable intermediate (2-amino-3-carboxymuconic acid-6-semialdehyde: ACMS) that is present at an important crossroads in the KP. The KP favours the non-enzymic cyclisation of ACMS to quinolinic acid (QA) which is further metabolised to NAD^+^. Alternatively, ACMS can be decarboxylated by ACMS decarboxylase (ACMSD: also known as picolinic acid carboxylase) to 2-aminomuconic-6-semialdehyde (AMS). AMS either cyclises non-enzymically to picolinic acid (PA) or undergoes further metabolism to acetyl CoA. PA production, however, becomes relatively more substantial if AMS dehydrogenase becomes saturated with its substrate. Activation of ACMSD can induce NAD^+^ depletion: a phenomenon of particular importance for the domestic cat (*Felis catus)*, which is unable to utilise Trp for NAD^+^ synthesis because of the greatly enhanced expression of its ACMSD [6].

### Control of the kynurenine pathway

The KP is regulated primarily by the first enzyme(s): TDO and IDO1 and secondarily by KYNU and ACMSD. The importance of TDO in control of the KP under normal physiological conditions is illustrated by the ∼9- to 13-fold increase in plasma [Trp] following *tdo2* gene deletion in mice [7,8] with a consequent 17-fold accumulation of Trp in liver [8] and a ∼ 10-fold increase in brain [Trp] [9]. Deletion of the *IDO1* or *IDO2* genes makes little difference to brain [Trp] [9]. Upon immune activation, however, IDO1 assumes a greater quantitative significance in control of Trp availability (see [10] and references cited therein). TDO is regulated by a number of mechanisms: hormonal induction of new apoenzyme synthesis by glucocorticoid, substrate activation and stabilisation by Trp, cofactor activation by haem and feedback inhibition by NAD(P)H. Other hormones may also be involved and it appears that haem itself plays a role in glucocorticoid induction of the enzyme (see discussion in [3]). IDO1 is regulated positively by interferon-γ (IFN-γ) and certain other cytokines and negatively by NO and by levels of Trp above 100 μM [3].

Flux of free (non-albumin-bound) Trp down the KP is another determinant of KP activity and has been studied in rat hepatocytes by the group of C I Pogson and in humans by this author (see [3] and references cited therein). Thus, Trp oxidation by TDO in hepatocytes is primarily a function of free (non-albumin-bound) Trp, with liver [Trp] being significantly correlated with liver [Kyn]. In normal humans, flux of plasma free Trp down the KP progresses dose-dependently, with maximum TDO activation being observed with a dose of Trp (5.15 g/70 kg body wt) equivalent to ∼ 74 mg/kg.

Whereas most other KP enzymes play their respective roles in control of the KP, some are of special interest because of their positions in the pathway: KAT, KMO, KYNU and ACMSD. KAT determines the production of KA, which is dependent on [Kyn] as the affinity of the enzyme for this substrate is low [3]. KMO is the gateway to production of further KP metabolites. KYNU plays a similar role, if less dramatically and is shown in hepatocyte studies to be the second rate-limiting KP enzyme after TDO. ACMSD acts at a crossroads in the pathway and its activity, also rate-limiting, determines the progress of the KP to eventual formation of NAD^+^.

The KP is also regulated by some if its intermediates and by body systems (see discussion in [11]). Thus, whereas studies *in vitro* suggested that, other than by NAD(P)H, TDO activity of *Xanthomonas pruni* or rat liver is inhibited by Kyn, 3-HK and 3-HAA, these findings could not be reproduced in rats after metabolite administration. Thus, using product/substrate ratios as indirect measures of enzyme activities, 3-HK does not alter TDO or KMO activities, but inhibits kynureninase activity, whereas 3-HAA stimulates TDO but inhibits KMO and kynureninase activities [12]. KP enzymes and metabolites can also be influenced by body systems, such as the endocrine and haematopoietic systems and by the major dietary staples [11].

### Pathways of NAD^+^ synthesis

**Figure 3:**
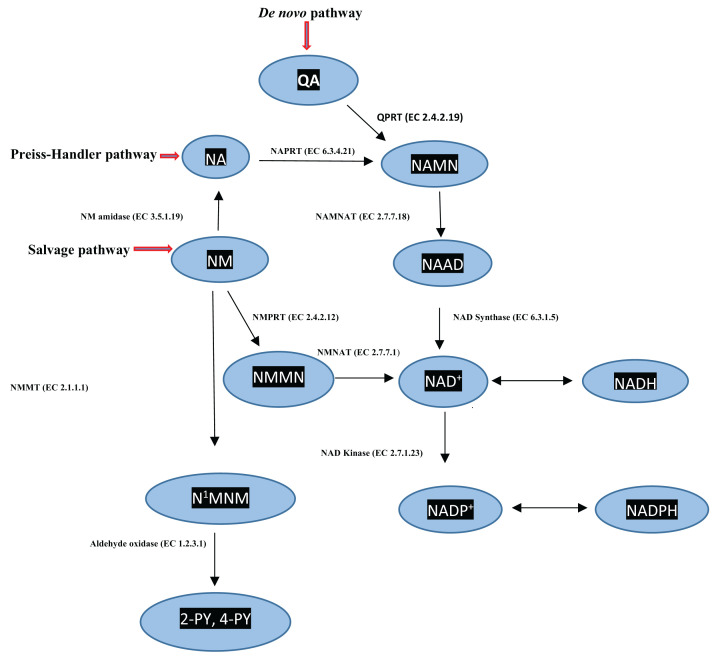
NAD^+^ synthesis from the *de novo*, Preiss-Handler and Salvage pathways Adapted here from Figure 3 in [2] by Badawy, A.A.-B. (Tryptophan metabolism and disposition in cancer biology and immunotherapy. *Biosci. Rep*. **42**, 1–27. BSR20221682 https://doi.org/10.1042/BSR20221682). Abbreviations: NA, nicotinic acid; NAAD, nicotinic acid-adenine-dinucleotide; NAD^+^(P^+^)H, [oxidized and reduced nicotinamide-adenine dinucleotides (phosphates)]; NAMN, nicotinic acid mononucleotide; NAMNAT, nicotinic acid mononucleotide adenylyltransferase; NAPRT, nicotinic acid phosphoribosyltransferase; N^1^MNM, N^1^-methylnicotinamide; NM, nicotinamide; NMMN, nicotinamide mononucleotide; NMNAT, nicotinamide mononucleotide adenylyltransferase; NMPRT, nicotinamide phosphoribosyltransferase; 2- and 4-PY, 2- and 4-pyridone carboxamides; QA, quinolinic acid.

NAD^+^ is synthesised by three pathways (Figure 3). In most mammals, the *de novo* biosynthetic pathway from QA predominates, with activity of quinolinate phosphoribosyltransferase (QPRT) being rate-limiting. The other two pathways, the ‘Preiss-Handler’ pathway from nicotinic acid and the ‘Salvage’ pathway from nicotinamide, are of minimal quantitative importance under normal physiological conditions, but could be activated under certain pathological conditions, e.g. viral infections and cancer.

The liver contains all enzymes of the KP leading to eventual formation of NAD^+^. The liver is also the major site of most of these enzymes [3]. By contrast, other tissues and cells do not possess the full complement, such that the number of pathway intermediates is restricted. Whereas TDO exists almost exclusively in liver and to a much lesser extent in brain, IDO1 is widely distributed throughout body organs and tissues. Notably, sites rich in IDO1 are the lungs, spleen, epididymis, intestines and immune cells. Only a few detailed systematic studies of KP enzymes in immune cells have been performed. Thus, Murakami and Saito [13] demonstrated in human cells considerable enzyme activities for IDO1, KMO, KYNU, and 3-HAAO in macrophages and monocytes, and of IDO1 in B-lymphocytes. Similarly, Belladonna et al. [14] demonstrated activities of the above four enzymes in mouse dendritic cells. Immunohistochemical studies can best identify immune and other cell types that accumulate large and measurable amounts of metabolites. The group of J R Moffett (see [15] and references cited therein) demonstrated accumulation of QA as a marker of KP enzymatic activities in immune cells, especially phagocytes and antigen-presenting cells (monocytes/macrophages, dendritic cells, microglia, Langerhans and Kupffer cells). It is important to note that, even if IDO1 is absent or poorly expressed in certain immune cells, the Kyn transported into them can be metabolised to QA, especially as cytokines such as IFN-γ and interleukin IL-1β also up-regulate KMO [16–19].

### Immune activity of metabolites of the pathway

Several KP metabolites are immunomodulatory. 3-HK, 3-HAA and QA are proinflammatory, whereas PA and possibly also AA are antiinflammatory. KA possesses both properties [20,21], hence its description as ‘Janus-faced’ [20]. Earlier studies showed that PA and QA act as immune signaling agents and 3-HAA as inhibitor of NO synthase expression (for references, see [22]), with NO being a negative effector of IDO1 [3]. Subsequently, 3-HK and 3-HAA were shown to suppress allogeneic T-cell proliferation in an additive manner and by an apoptotic mechanism and, additionally, 3-HAA and QA were shown to undermine T-helper Th1 cells, resulting in a shift of the balance towards Th2 [23,24].

Certain Trp metabolites also activate the aryl hydrocarbon receptor (AhR), which is a transcription factor that can elicit destructive as well as protective effects [25]. Destructive effects occur after AhR activation by exogenous chemicals, the best known of which is TCDD (2,3,7,8-tetrachlorodibenzo-*p*-dioxin). Endogenous ligands of the AhR are generally associated with protective effects, though excessive activation can cause harm. Endogenous ligands include a range of Trp metabolites. At 10 µM, KA possesses the highest AhR stimulatory effect among KP metabolites, with XA exhibiting only 17% of the KA potency, whereas the other metabolites Kyn, 3-HK, QA and AA exert no significant effect at this concentration [26]. Indole metabolites of Trp produced in other degradative pathways and by gut microbiota are also AhR ligands [27,28], with the Trp transamination product indol-3ylpyruvic acid (IPA) being a more powerful AhR activator than KA [29]. Another Trp-derived compound is 6-formylindolo (3,2-b) carbazole (FICZ), a Trp photo-oxidation product and a metabolite formed via IPA [30,31]. Both KA and FICZ exert important effects on immune function that can undermine immune defences against infection, possibly including that by COVID-19. The AhR controls IDO1 expression via AhR-IL-6-STAT3 signaling [32,33] and although KA induces IL-6, the elevation of this cytokine by inflammation can induce IDO to produce sufficient amounts of KA to activate the AhR [26]. The recently identified metabolic immune checkpoint IL-4I1 (IL-4-induced gene 1) (*L*-phenylalanine oxidase) activates the AhR via KA and IPA [29], with the latter possibly also acting in part by conversion to KA via an unstable intermediate (see [34]). The importance of KA in the AhR activation is further suggested by IL4I1 increasing [KA] but not [Kyn] [29]. Elevation of KA levels occurs in plasma and other body fluids and in cell supernatants from inflammation-related conditions and some cancers (see [34] for references). Thus, whereas elevation of Kyn levels by viral and other infections initiates the AhR activation, this latter process is most likely to be mediated by the KA produced through KAT activity. This is because [Kyn] provides the impetus for the KAT reaction in view of the low affinity of the enzyme for Kyn (*K*_m_ = 0.96–4.7 mM) [3]. An increase in [Kyn] can activate the KAT reaction by the law of mass action without the need for KAT activation, induction or enhanced mRNA gene expression. Invariably, the increases in [Kyn] and [KA] after a Kyn elevation do not alter the [KA]/[Kyn] ratio. Accordingly, the absence of an increase in this ratio should not preclude an enhanced KAT reaction. IL-6 along with IL-1α and IL-1β exhibit enhanced expression by FICZ [35]. FICZ plays important roles in many biological processes [31]. Regarding inflammation, a distinction between TCDD and FICZ is their opposite effects on differentiation of immune cells, with TCDD inducing regulatory T cells (T_reg_) and FICZ interfering with T_reg_ development, but promoting differentiation of proinflammatory Th17 cells [36,37].

The AhR also controls the gene expression of poly (ADP-ribose) polymerases (PARPs) [29,38]. PARPs catalyse the transfer of adenosine diphosphate (ADP)-ribose to target proteins, thereby influencing many important cellular processes. PARPs are NAD^+^ consumers, with PARP1 alone consuming ∼33% of total cellular NAD^+^ [39]. Whereas PARP activation is not intrinsically harmful, over activation can be, with PARP 1 over-activation resulting in cell death involving in part depletion of NAD^+^ and ATP [40,41].

Thus, a disturbed Trp metabolism involving increased proinflammatory activity of KA and possibly also FICZ and mediated by activation of the AhR and PARPs are likely to be at the centre of the pathophysiology of infectious diseases, including COVID-19. It is important to emphasize that increased production of proinflammatory and other Kyn metabolites is determined not only by TDO or IDO induction, but, additionally, by the flux of plasma free Trp down the KP. Free Trp levels are determined by extent of binding by albumin and of displacement by non-esterified fatty acids (NEFA) [3]. Infection is associated with a low circulating [albumin] and a raised [NEFA], the influence of which on production of Kyn metabolites in inflammatory conditions, including COVID-19, should be assessed. Additionally, regarding NEFA, AhR induces various changes in lipid metabolism, including decreased fatty acid oxidation and export and increased mobilisation and import, all resulting in increased hepatic [NEFA] and triacylglycerols leading to fatty liver production [42,43]. AhR activation can therefore contribute to increased flux of plasma free Trp down the KP.

## The immune status in COVID-19

Studies of the immune status of SARS-CO-V-2 (COVID-19) are summarised in Table 1 [44–53]. Early studies showed that immune function is negatively impacted by this coronavirus infection. In studies with small samples, a proinflammatory environment prevailed in patients requiring intensive care [44] that was associated with viral load and lung pathology [45]. Two subsequent longitudinal studies in larger samples identified early markers of disease outcome in clusters [46] or signature traits [47], that could act as predictors of coagulopathy, length of hospitalisation, severity of infection and death, with interleukins IL-6, IL-8, IL-10, IP-10 (IFN-γ-induced protein 10), TNF-α and IFN-α featuring prominently among these predictors. The summary in Table 1 lists the increases in cytokines and chemokines associated with severity of COVID-19 infection that reflect a predominance of proinflammatory cytokine elevation. The list in Table 1 is not comprehensive, but is of sufficient detail to illustrate the elevation of proinflammatory cytokines in COVID-19. In studies with other respiratory viral infections (SARS, MERS, RSV and COPD) a broadly similar picture of elevated levels of IL-6, IL-8, IL-12, IL-17, TNF-α, IFN-α and IFN-γ has been observed (see [34] for references).

**Table 1:** Cytokine and chemokine changes in COVID-19

Elevation of plasma proinflammatory cytokines and chemokines	[Ref]
IL-2, IL-7, IL-8, IL-12, IL-17	[44]
M-CSF, IL-10, IFN-α2, IL-17, IL-4, IP-10, IL-7, IL-1ra, G-CSF, IL-12, IFN-γ, IL-1α, IL-2, HGF and PDGF-BB	[45]
IL-1α, IL-1β, IL-2, IL-4, IL-5, IL-12 p70, IL-13, IL-15, IL-17A, IL-17E, IL-22, IFN-γ, TNF-α, CCL1, CCL2, CCL5, CCL8, CCL15, CCL21, CCL22, CCL27, CXCL9, CXCL10, CXCL13, SDF1 (CXCL12)	[46]
IL-6, IL-8, IL-10, IP-10 (IFN-γ-induced protein 10), TNF-α, IFN-α	[47]
IL-2, IL-4, IL-6, IL-12, IFN-α-2a, IFN-β, IFN-γ, TNF-α, CCL4, CCL5, CCL10, CCL11, CXCL11	[48]
CTACK, G-CSF, GM-CSF, GRO-a, HGF, IFN-α2, IFN-γ, IL-1α, IL-1β, IL-2ra, IL-5, IL-6, IL-7, IL-8, IL-9, IL-10, IL-12(p70), IL-12(p40), IL-13, IL-15, IL-17, IL-18, IP-10, LIF, M-CSF, MIF, MIG,β-NGF, SCGF-β, SDF-1a, TNF-α, TNF-β, VEGF	[49]
IL-1ra, IP-10, SDF-1α, IL-8, TNF-α, MIP-1β, MCP-1	[50]
IL-1β, IL-6, IL-10	[51]
IL-1β, IL-6, IL-10, IL-18, IFN-γ	[52]
IL-6, CXCL9, CCL2	[53]

Abbreviations: β-NGF, β-nerve growth factor; CCL, chemokine C–C motif ligand; CSF, colony-stimulating factor; CTACK, cutaneous T-cell-attracting chemokine; CXCL, chemokine C-X-C motif ligand; GM-CSF, granulocyte colony stimulating factor; HGF, hepatocyte growth factor; IFN, interferon; IL, interleukin; IL-12 (p40), interleukin; IL-12 p40 subunit; IL-12 (p70), IL-12 p70 subunit; IP-10, interferon-γ-inducible protein; LIF, leukemia inhibitory factor; MCP, monocyte chemoattractant protein; M-CSF, macrophage colony stimulating factor; MIF, macrophage migration inhibitory factor; MIG, monokine-induced by interferon-γ; MIP, macrophage inflammatory protein; PDGF-BB, platelet-derived growth factor with 2 (BB) subunits; SCGF-β, stem cell growth factor β; SDF-1α, stromal cell-derived factor 1α; TNF, tumor necrosis factor.

## Potential impact of immune and metabolic changes on tryptophan metabolism in COVID-19 infection

From the above accounts, a number of factors can modulate Trp metabolism along the KP in COVID-19 patients. These are outlined in Table 2, which also gives their status in this infection.

**Table 2:** Determinants and mechanisms of changes in kynurenine and other metabolites in COVID-19 infection

Determinant	Feature	Effect(s)/Mechanism	[Ref]
Immune activation	↑ PI cytokines	Inflammation	[44–53]
	↑ IDO1	↑[Kyn]/[Trp]	[52,53,60–73]
↓ Plasma albumin	*↑ [Free Trp]*	↑ [Kyn metabolites]	[77–81]
↑ Plasma NEFA	*↑ [Free Trp]*	↑ [Kyn metabolites]	[61,81–84]
↑ Plasma cortisol	*↑ TDO*	↑ [Kyn metabolites]	[66,68,89–92]
↑ KP metabolites	↑ Kyn	↑ IDO1, *↑ TDO, Trp Flux*	[52,54,60–73,108]
	↑ KA	↑ Kyn (*KAT A activation*)	[60,61,68,70,106]
	↑ 3-HK	↑ Kyn (*KMO activation*)	[57,66,70,107]
	↑ QA	?	[49,70,107]
	↑ AA	(*KYNU activation*)	[107]
	↑ PA	?	[61,70]
	↑ NA	?	[50,61]
↓ KP metabolites	↓ Trp	↑ IDO1, *↑ TDO*	[52,53,60–73]
	↓ XA	*↓ KAT B*	[50]
	↓ AA	*↓ KYNU*	[61]
	↓ 5-HT	↓Trp (↑ IDO1, *↑ TDO*)	[50,61,108]
	↓ IAA	↓Trp (↑ IDO1, *↑ TDO*)	[43]
	↓ IPA	↓Trp (↑ IDO1, *↑ TDO*)	[61]
↑ AhR	↑ IDO	↑ IFN-β, ↑ IFN-γ	[109–111]
	↑ PARP		[112]
↑ PARPs	↓ NAD^+^, ↓ ATP	↑ AhR, DNA damage	[113,114]
↓ SIRTs	↑ ROS, ↑ Cytokine storm	↑ PARP, ↓ NAD^+^, NAM	[103,127]

Symbols: ↑ increase, ↓ decrease, ? untested. Abbreviations: 3-HK, 3-hydroxykynurenine; 5-HT, 5-hydroxytryptamine (serotonin); AA, anthranilic acid; AhR, aryl hydrocarbon receptor; ATP, adenosine triphosphate; IAA, indol-3-yl-acetic acid; IDO, indoleamine 2,3-dioxygenase; IFN, interferon; IPA, indolepyruvic acid; KA, kynurenic acid; KAT, kynurenine aminotransferase; KMO, kynurenine monooxygenase; Kyn, kynurenine; KYNU, kynureninase; NA, nicotinic acid; NAD^+^, oxidised nicotinamide-adenine dinucleotide; NEFA, non-esterified fatty acids; PA, picolinic acid; PARP, poly (ADP-ribose) polymerase; QA, quinolinic acid; ROS, reactive oxygen species; SIRT, sirtuin; TDO, Trp 2,3-dioxygenase; Trp, *L*-tryptophan. Lettering in italics indicates likely, but as yet, undemonstrated changes.

### Immune activation and changes in tryptophan and kynurenine metabolite levels

The increased release of the above proinflammatory cytokines in COVID-19 patients is likely to induce IDO1 activity leading to accelerated Trp degradation along the KP. Interferons (IFNs) were the first proinflammatory cytokines reported to induce IDO activity, with IFN-γ being a stronger inducer of enzyme activity and expression than IFN-β > IFN-α [54–56]. Other proinflammatory cytokines, notably IL-1β and TNF-α can also induce IDO1 but act mainly through IFN-γ [57]. TNF-α can also induce IL-6 gene expression [58]. As stated above, IL-6 can also participate in IDO induction through the AhR-IL-6-STAT3 autocrine loop. Proinflammatory cytokines can also modulate expression of other KP enzymes. Thus, IL-1β and IFN-γ enhance the gene expression of KMO [13,24–26]. KYNU expression is also enhanced by IFN-γ [17,19,59].

That the KP is activated by COVID-19 is evident from 16 studies [52,53,60–73] demonstrating an increase in the [Kyn]/[Trp] ratio. Of these 16 studies, 2 did not provide individual data on [Kyn] or [Trp], one provided Trp only data, 2 provided Kyn only data and 11 provided data for both parameters. In most of these latter studies, cytokine levels were not measured, though in two [65,66] severity or death was correlated with IL-6 levels. The study by Lionetto et al. [64] has assessed the [Kyn]/[Trp] ratio in greater detail. The increased ratio was more prominent in males and correlated with age and severity of infection. Also, the largest increase in the ratio was observed in patients with severe lymphopenia [70]. Lymphopenia is observed in association with raised cortisol levels in SARS coronavirus (CoV) before glucocorticoid therapy, which appears to complicate its consequences [74]. Cihan et al. [70] also measured various kynurenine metabolites and, from the observed increases (described in Table 2), it is possible to assess indirectly changes in KP enzyme activities with progress of infection from control to moderate to severe infection to that requiring intensive care. The most remarkable changes were in the [Kyn]/[Trp] ratio (increases above controls of 23%. 100% and 132% respectively) and in the [QA]/[3-HK] ratio of 223%, 260%, and 321% respectively. There were also increases in the [KA]/[Kyn] ratio of 122%, 44%, and 53% respectively, whereas the [3-HK]/[Kyn] ratio was decreased by 5%, 26%, and 28% respectively. The most remarkable increases in KP metabolites were 183% rise in [KA] and the 464% rise in [QA] [70].

The increase in plasma [Kyn] and the decrease in [Trp] in the above studies varied between 20–150% and 12–50% respectively. Except in ‘closed’ *in vitro* culture systems, the Kyn elevation and the Trp decline are not a usual feature of IDO1 induction *in vivo*, which is invariably associated with a decrease in [Trp], with [Kyn] remaining unaltered or slightly increased [75]. This suggests that, in COVID-19 infection, another mechanism(s) is active in parallel with IDO induction. Potential mechanisms are TDO induction by cortisol, increased flux of plasma free (non-albumin-bound) Trp down the KP or inhibition of KMO or KYNU [75]. The potential roles of these determinants will be explored below.

### Albumin depletion and NEFA elevation in COVID-19 as determinants of flux of plasma free tryptophan down the KP

The effect of COVID-19 on plasma [free Trp] is unknown. That its flux down the KP is likely to be enhanced in COVID-19 is strongly suggested by the reported decrease in plasma [albumin] and increase in [NEFA]. [Albumin] is significantly decreased in severe cases of COVID-19 patients, including those requiring intensive care, by 10–20% and is considered an independent death risk factor linked to serious effects of the virus, such as coagulopathy, vascular disease and lung injury [44,76–79]. A decrease in plasma [albumin] of ≥20% is likely to cause the release of Trp from its binding sites [80].

An increase in Plasma [NEFA] in COVID-19 patients is correlated with progress of the infection [61,81–83]. As discussed above, AhR activation can contribute further to the NEFA elevation. As simultaneous albumin depletion and NEFA elevation provide the optimum impact on plasma Trp binding to albumin, increased flux of free Trp down the KP can therefore be expected to enhance KP metabolite formation irrespective of TDO/IDO induction. Measurement of plasma [free Trp] along with albumin and NEFA will therefore be an important requirement in future studies of COVID-19 pathophysiology. Indirect evidence for elevation of plasma free Trp in COVID-19 is provided from the salivary data in severe cases reported by Sharif-Askari et al. [73] showing a ∼ doubling of salivary [Trp] associated with a ∼50% decrease in plasma levels. Associated with these opposite changes in [Trp] were remarkable increases in [Kyn] in both plasma (3-fold) and saliva (12-fold). As Trp degradation in COVID-19 involves IDO1/TDO induction, the rise in salivary [Trp] can only reflect increased availability of the amino acid, arising most likely from a potential free plasma Trp elevation. The relationship between salivary and plasma free [Trp] has not been investigated, but a parallel exists for plasma free and salivary cortisol. Thus, both plasma free [84] and salivary [85] cortisol are ∼ 10% of plasma total cortisol. With a plasma free and total [Trp] in normal subjects (*n*=114) of 5.29 and 63 μM respectively [5] (1.08 and 12.87 μg/ml respectively), the reported salivary [Trp] values of 0.4–0.85 μg/ml [86,87] are most likely a reflection of plasma free, rather than total, Trp. That the above 3-fold increase in plasma [Kyn] is accompanied with a 13-fold increase in salivary levels [73] suggests that salivary Kyn also reflects plasma free Kyn, which is ∼ 20% of total plasma [Kyn] [88].

### Potential glucocorticoid induction of TDO in COVID-19

While TDO activity or expression has not been studied in COVID-19 patients or experimental models, cortisol levels are raised in severe cases including fatal ones [66,68,89,90]. In another study [91], low cortisol levels were initially reported, but were not reproducible a few days later. Doubling cortisol levels increased mortality by 42% [92]. Under these conditions, it is almost certain that TDO will be induced in patients with raised cortisol levels, thus contributing to KP metabolite formation. A glucocorticoid receptor associated specifically with TDO induction, but not that of Tyr aminotransferase, is elicited by stress in rats [93,94]. Glucocorticoid receptor function could therefore be explored in future COVID-19 studies.

### Lymphopenia and lung pathology

The relationship between glucocorticoids, lymphopenia and COVID-19 pathology is worthy of discussion. Incidence of lymphopenia is more frequent in severe COVID-19 patients requiring intensive care [44,95] and cortisol levels are raised in SARS patients with lymphopenia [74]. Lymphopenia in association with neutrophilia are features of glucocorticoid excess and are caused by glucocorticoids enhancing lymphocyte migration from peripheral blood and inhibition of apoptosis [96]. As well as by raised cortisol levels, various direct and indirect mechanisms have been proposed to explain the effects of COVID-19 on lymphocytes. These include destruction of lymphocytes, the cytokine storm, impaired adhesion of vascular cells, decreased numbers of natural killer, B and T cells, inhibition of T-cell immunity by IL-6, increased lactate levels, and associated comorbidities influencing endothelial function (see [97] and references cited therein). Transcriptome data prediction and reuse show that, in bronchoalveolar gavage fluid of COVID-19 patients, there are significant major increases in neutrophils and activated mast cells [98,99]. COVID-19 targets mainly alveolar type II cells wherein cell death and a strong innate immune response contribute to acute respiratory distress [100]. These and other aspects of immune dysfunction [101–103] illustrate the complexity of effects of COVID-19 on the immune system that are outside the immediate scope of this Review. Decreased SIRT1 expression and an imbalance of the P53/SIRT1 axis have been proposed as determinants of lymphocyte dysfunction [103]. Neutrophils are by far the largest circulating blood cells and while paradoxically they are the first line of innate immune defence, they are also mediators of inflammation [104]. COVID-19-infected lung cells up-regulate six chemokines of the neutrophil chemotaxis type that include a range of neutrophil chemoattractants and C3 and pathway activation genes that are associated with lung neutrophilia (see [105] and references cited therein).

In relation to Trp metabolism, the heightened immune activation of epithelial cells [100], the release of proinflammatory cytokines from virus-infected macrophages and dendritic cells [101] and the further recruitment of these cells by neutrophils are almost certain to result in a strong localised IDO induction leading to increased production of T-cell apoptotic Kyn metabolites and associated oxidative stress, PARP1 activation, NAD^+^ depletion and SIRT1 down-regulation (see below). That the apoptotic 3-HAA and QA accumulate in lung interstitial and endothelial cells and in types I and II pneumocytes has been demonstrated by Guo et al. [72]. These latter authors also showed that these Kyn metabolites co-stained largely with IDO2 and minimally with IDO1. Lung cells expressing IDO2 also co-stained with markers of autophagy (LC3B) and apoptosis (cleaved caspase 3).

Some KP enzymes and metabolites are also upregulated in COVID-19 in the nasal cavity: the gateway to the respiratory tract, the primary site of COVID-19 infection. Thus, nasal swabs showed increased levels of Kyn, KA, AA, 3-HAA, QA and PA and upregulation of IDO and KYNU [106]. These latter authors [106] also noted that KP gene expression associates with increased inflammation and severe disease outcome, thus adding further support to the earlier finding [61] that the KP is the top pathway targeted by COVID-19. IDO induction and increased production of excitotoxic Kyn metabolites have also been proposed as mediators of the COVID-19-induced anosmia [107]. The detection and demonstration of increased production of Kyn metabolites in the nasal cavity suggest that enzymes of the KP up to and including ACMSD are actively expressed in this anatomical structure.

### Changes in KP metabolites and enzymes beyond IDO and TDO

Whereas most studies of the KP in COV1D-19 focused on changes in plasma Trp and Kyn levels, only a few studies extended observations to other Trp metabolites. As shown in Table 2, the most frequent changes reported are the increases in plasma KA and 3-HK and the decrease in serotonin (5-HT). The latter can simply be attributed to decreased Trp availability for Trp hydroxylase (TPH). The huge increase in [KA] of 129-183% [68,70,108] is likely to be the result of increased precursor concentration, because of the low affinity of KAT for Kyn [3]. Thus, increased Kyn availability overrules the need to upregulate KAT in order to raise [KA]. The simultaneous increase in [3-HK] would not normally be expected if KAT activity is enhanced by other than increased substrate availability, but it could be explained by the abundance of the Kyn substrate for both KAT and KMO given the greater affinity of KMO for Kyn [3]. Although in the study by Cihan et al. [70] [3-HK] was elevated, KMO activity expressed as the [3-HK]/[Kyn] ratio was in fact decreased. As stated above, the increase in [Kyn] could be as large as 150%, which is likely to involve factors other than IDO1 induction: perhaps additionally by TDO induction and/or increased flux of plasma free Trp down the KP. The huge plasma QA elevation despite the apparent KMO inhibition [70] suggests that host QA metabolism to NAD^+^ may be inhibited in COVID-19: perhaps by decreased quinolinate phosphoribosyltransferase (QPRT). The small number of observations of changes in other KP metabolites in Table 2, however, requires confirmation in future studies before they could be adequately interpreted.

A prominent feature of the changes in the KP metabolites listed in Table 2 is the importance of the KA elevation in the severity of COVID-19 infection in association with the widely observed increase in [Kyn]. Severity was correlated strongly with levels of Kyn, 3-HK and KA [45,108]. In the latter study [108], the [KA]/[Kyn] ratio reflecting the KAT reaction correlated with levels of IL-6, CCL1, CCL21, TNF-α and M-CSF. Analysis by these authors [108] of Genotype-Tissue Expression (GTEx) data showed that KAT expression correlates most strongly with cytokine levels and AhR activation in older males. Trp metabolism via the KP was identified in targeted and untargeted metabolomics data as the top pathway affected by COVID-19 [61] with gene expression of the pathway enzymes being a major factor in severe disease outcome [106]. Other metabolic pathways targeted by COVID-19 are those of lipid metabolism, glucose, energy and oxidative metabolism, urea cycle and kidney function [61] Table 3). In this latter study, the changes in these pathways correlated with markers of inflammation (IL-6 and C-reactive protein) and/or renal function (blood urea nitrogen). COVID-19 targets also other (non-metabolic) pathways, such as the immune system, angiotensin-converting enzyme ACE2 and others. Taken together, the above findings in the KP of Trp metabolism suggest that, of the metabolites studied, KA plays an important role in COVID-19 pathophysiology. A potential impact of KA is its activation of the AhR.

**Table 3:** Pathways targeted by COVID-19

Pathway	Main changes
1. Amino acids	
Trp	Activation of the kynurenine pathway and modulation of activities of its enzymes (see Table 2)
Other AAs	Various changes
2. Lipid metabolism	Increased circulating fatty acids (→ raised plasma free Trp)
3. Oxidative metabolism	Increased glycolytic and pentose phosphate pathways Increased markers of oxidative stress
4. Kidney function	Increased creatine, creatinine and polyamines

Based on findings by Thomas et al. [61].

## Role of AhR activation in COVID-19 pathophysiology

The AhR is activated in COVID-19 infection [111–114]. Liu et al. [111] reported up-regulation of mucins in lungs secondarily to IFN-β or IFN-γ up-regulating the AhR by an IDO-Kyn dependent pathway in SARS-CoV-2 infected mice and macaques and suggested that accumulation of mucins in alveolar epithelial cells may explain the virus-associated hypoxia. In this experimental study [111], the [Kyn] was too high (400 µM) to be achieved under *in vivo* pathological conditions in COVID-19, wherein the maximum reported increase in plasma levels was 1.5-fold above the normally low µM basal values. As [Kyn] at 10 µM does not activate the AhR [26], it is most likely that AhR activation in COVID-19 is mediated largely by KA. Whereas COVID-19 infection can activate the AhR by an IDO-independent mechanism, the AhR can enhance its own activity by controlling IDO expression via the above autocrine loop [32,33]. It is possible that coronavirus infection activates the AhR in an IDO-independent manner, contributing to cytokine modulation and proviral TCDD-inducible-PARP expression [114].

## Role of PARP activation in COVID-19 pathophysiology

Several TCDD-inducible or MARylating (transferring only one moiety of ADP-ribose) PARP and also IDO1 genes are indeed up-regulated in COVID-19 [115]. MARylating PARPs are PARPs 3, 4, 6–12 and 14–16, whereas PARylating PARPs (Transferring many ADP-ribose moieties) are PARPs 1, 2 and 5. Thus, PARPs 9, 12 and 14 are induced in enterocyte organoids infected with SARS-CoV-2 and, in infected ferrets, PARPs 4, 5, 9, 13, 14, and 15 are 4-fold induced with significant but lesser induction of PARPs 7 and 11 [115]. PARPs 9, 11, and 13 are up-regulated in the lung of a deceased COVID-19 patient [116], and in broncho-alveolar gavage fluid of infected patients, PARP 9 is up-regulated by ∼ 4-fold whereas PARPs 12 and 14 are up-regulated by ≥ 2-fold [115]. These latter authors also demonstrated that expression of genes encoding the salvage pathway of NAD^+^ synthesis from nicotinamide or its riboside is induced by COVID-19, whereas NAD^+^ synthesis from other pathways catalysed by NAD^+^ synthase is down-regulated. A similar strategy is adopted by a wide range of cancers [117–119]. The virus thus demonstrates a cleverness in blocking the major and main route of NAD^+^ synthesis in host and facilitating the ‘salvage’ pathway to maintain its own NAD^+^ metabolome. Heer et al. [115] also demonstrated that over expression of PARP 10 alone is sufficient to deplete cellular NAD^+^ by > 90%.

PARP1 is the major PARP and consumes about 33% of cellular NAD^+^ [39]. Although in the study by Heer et al. [115], PARP 1 *gene expression* was not enhanced by COVID-19, activation of PARP 1 in COVID-19 patients cannot be entirely ruled out, given the cytokine-induced DNA damage [120,121], with the expected greater consumption and hence depletion of NAD^+^ [122,123]. PARP 1 activation is an important feature in patients with, or experimental models of, lung diseases [122–128].

## Down regulation of Sirtuin 1 by COVID-19

The other major NAD^+^ consumer is sirtuin 1 (silent mating type information regulation 2 homologue 1), which also consumes ∼33% of cellular NAD^+^ [39]. By contrast with the up-regulated PARPs, SIRT 1 is down-regulated in COVID-19 in peripheral blood mononuclear cells [103], and lung epithelial and endothelial cells [129]. At least three mechanisms can explain the low SIRT 1 in COVID-19. As both PARPs and SIRTs compete for the same (NAD^+^) substrate, up-regulation of PARPs inhibit SIRT 1 through diminished NAD^+^ availability. The enhanced PARP reaction increases the product nicotinamide, which is a potent SIRT 1 inhibitor at physiological concentrations (see [34] for discussion and references). Thirdly, SARS-CoV-2 inactivates SIRT 1 via the non-structural viral protein NSP14, which interacts with its catalytic domain [130]. The latter authors suggested that SIRT 1 protects against the cytokine storm and oxidative stress induced by SARS-CoV-2 by activating the nuclear erythroid 2-related factor 2/haem oxygenase 1 (NRF2/HMOX1) complex, which acts via the HOX reaction product biliverdin. An additional potential SIRT 1-related protective mechanism is that HOX1 activation depletes haem levels, resulting in impaired liver TDO activity [131], thereby potentially preventing production of proinflammatory Kyn metabolites. Down-regulation of SIRT 1 in COVID-19 is associated and highly correlated with elevated levels of IL-1β, IL-6, IL-8, and TNF-α [103]. Decreased levels of SIRTs 1 and 3 also correlate with severity of skin and lung fibrosis and peripheral microvasculopathy in systemic sclerosis [132]. Thus, it is most likely that SIRT 1 depletion results mainly from PARP activation induced by the AhR, activation of which may be mediated by KA in conjunction with proinflammatory cytokines.

## The kynurenine pathway and the brain in COVID-19

The COVID-19-induced dysfunction of the KP could influence brain function in many ways, notable among which are disturbed mood, cognitive deficits and neurotoxicity. Anxiety and depression can arise in part from a serotonin deficiency, whereas impaired cognition and neurotoxicity can be induced by glutamatergic activation. Both mechanisms operate in COVID-19 and may underpin these behavioural disorders [133,134]. Serotonin deficiency in the periphery is suggested by the decrease in plasma [5-HT] [50,61,108], and, although peripheral [5-HT] bears no relation to cerebral levels, the decrease in plasma suggests a shortage of availability of the Trp precursor, almost certainly due to accelerated Trp degradation by induction of IDO1, TDO2 or both. A central serotonin deficiency is determined by a decrease in plasma Trp availability to the brain. Availability is influenced in part by extent of competition with Trp from the large neutral (competing) amino acids (CAA) Val, Leu, Ile, Phe and Tyr. The [Trp]/[CAA] ratio is the best indirect indicator of Trp entry into the brain and consequent serotonin synthesis. Various studies have demonstrated the decrease in plasma [Trp] (Table 2), but very few have reported data on the 5 CAA and none has addressed the [Trp]/[CAA] ratio. Only three studies in which concentrations of both Trp and the five competitors were measured have been published [71,135,136] from which the sums of CAA and ratios were calculated by this author (Table 4). The [Trp]/[CAA] ratio was decreased in all 3 studies by 46%, 53%, and 49% respectively. The decrease in this ratio was due largely to the 32–47% decrease in [Trp] in two studies [71,135], and a 90% increase [CAA] in the third study [136], despite the serum having been obtained from fasting blood in this latter study. A decrease in the above ratio of this magnitude is sufficient to reflect decreased entry of Trp into the brain and a consequent inhibition of 5-HT synthesis. A central 5-HT deficiency can also arise within the brain if Trp is depleted by local induction of IDO (or TDO, which also exists in brain). Such induction is, however, unsupported by current evidence, as little work has been done to investigate cerebrospinal fluid (CSF) levels of Kyn or serotonin metabolites or the cytokine status in COVID-19 patients. Thus, most CSF studies assessed certain immune parameters (immunoglobulins) in relation to neurological symptoms. Only 3 studies addressed markers of inflammation and/or neurotransmission. Inflammation was absent in one study [137], and present in another [138] and levels of glutamate, glutamine and GABA (γ-aminobutyric acid) were not different from normal in a small number of patients [134]. Clearly, more studies are required to assess a potential central serotonin deficiency initiated within the brain.

**Table 4:** The plasma tryptophan to competing amino acid ratio in COVID-19 patients

Study No.	Ref No.	[Trp]		[CAA]		[Trp]/[CAA]	
		Control	COVID	Control	COVID	Control	COVID
1	71	93	63	650	519	0.175	0.121
2	135	41	22	405	458	0.101	0.048
3	136	63	61	430	815	0.146	0.075

Numbers of patients and non-COVID controls were as follows: Study 1 (32 and 39, respectively); Study 2 (32 and 28, respectively); Study 3 (38 and 30, respectively). Data extracted and calculated from the three referenced papers.

Glutamatergic activation can however arise within the brain through entry of circulating Kyn and QA. Kyn enters the brain via the large neutral amino acid LAT and SLC transporters, whereas QA enters the brain by passive diffusion [139–141]. 3-HK is also transported by the LAT system [140]. As Kyn is the precursor of both KA and QA, increases in cerebral levels of these two NMDA receptor modulators can be expected in COVID-19 infection through their cerebral uptake or that of Kyn, or if brain IDO is induced to produce the Kyn precursor. CSF [QA] is strongly elevated in humans suffering from a range of bacterial and viral infections [142]. As the rise in QA was not associated with decreased [Trp] [142], transport of QA and/or its Kyn precursor, rather than cerebral IDO induction, is the more likely mechanism of the QA elevation. Heyes et al. [142] also demonstrated large increases in CSF [Kyn] and [KA] in these infections, though the QA elevation was greater than that of KA, leading to an increased [QA]/[KA] ratio that is consistent with glutamatergic activation in inflammatory disease. Although Fukui et al. [140] reported a low cerebral uptake of QA in rats, Heyes and Morrison [143] reported that 38–49% of brain [QA] in gerbil is of peripheral origin. Species differences are clearly likely and it remains to be seen how COVID-19 influences cerebral Kyn metabolite levels in humans.

The so-called ‘Long COVID’ is also associated with mood and neurological disorders, with a range of somatic and psychological symptoms having been reported [144]. The potential role of the KP in these symptoms has not been investigated, except in one recent study. A number of studies addressed potential markers or determinants of Long COVID [145–147]. One of which [146] reported that a proportion of patients exhibit an elevation of proinflammatory cytokines with a persistent low-grade inflammation and reporting of troubling symptoms. It is possible that this subgroup of susceptible individuals may suffer from a persistent KP dysfunction akin to that during acute infection. In the recent study by Cysique et al. [148], cognition was impaired over a 12-month post the acute COVID-19 period, at least once in 46% of patients, with elevation of Kyn, 3-HAA, QA, and the [Kyn]/[Trp] ratio being associated with poorer cognitive performance and greater likelihood of impairment. As the authors [148] reported no change in plasma [Trp], the above increases in KP metabolites are likely to have been caused by changes beyond TDO/IDO, possibly activation of KMO and/or KYNU. More studies of the KP during the long-term post-COVID-19 phase are clearly required.

## The kynurenine pathway is targeted by COVID-19 and should be the focus of its immunotherapy

**Figure 4:**
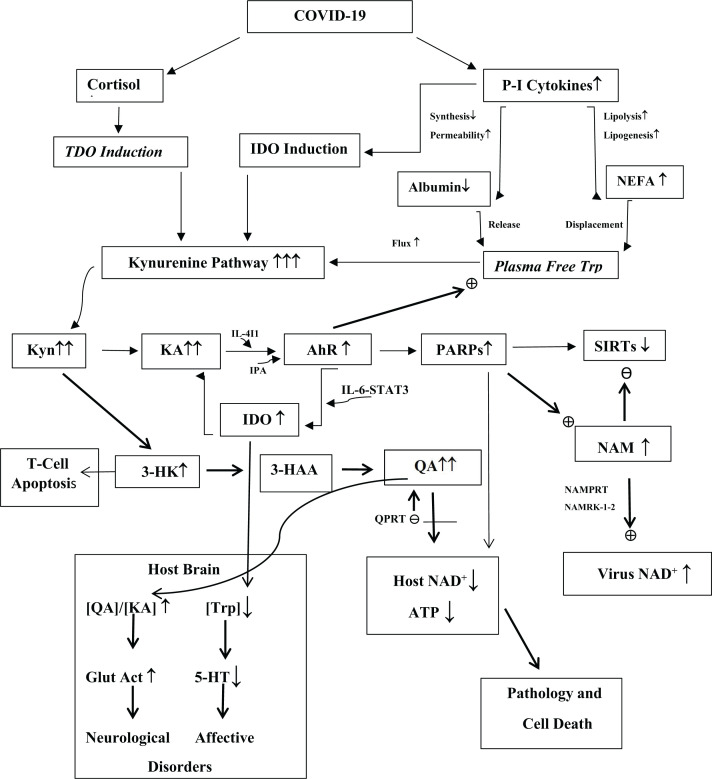
Changes in kynurenine pathway and related processes as potential determinants of COVID-19 pathophysiology This is an updated version of a previously published Figure [Figure 3 in ref [34] by Badawy A.A.-B. (*Biosci. Rep.* 2020; **40** BSR20202856 https://doi.org/10.1042/BSR20202856) Arrows between metabolites or other parameters indicate reactions or effects, whereas arrows next to metabolites and other parameters denote level of increase **↑** or decrease **↓.** Words in *italics* represent likely, but as yet, unmeasured changes (*TDO; plasma free Trp*). Abbreviations: Glut Act, glutamatergic activation; IPA, indol-3-ylpyruvic acid; NAM, nicotinamide; NAMPRT, nicotinamide phosphoribosyl transferase; NAMRK, nicotinamide riboside kinase; NEFA, non-esterified fatty acids; P-I, proinflammatory; QPRT, quinolinate phosphoribosyltransferase. Various aspects are presented in this Figure. Activation of the KP by COVID-19 is mediated by IDO induction by proinflammatory cytokines, potential TDO induction by cortisol and increased flux of plasma free Trp down the KP. The increased production of Kyn triggers several changes: production of the AhR agonist KA, which leads to PARP activation, SIRT depletion, increased virus NAD^+^, loss of host NAD^+^ and ATP and cell death. Production of Kyn metabolites undermines T-cell function thereby minimising host anti-viral defences. KP activation results in Trp depletion leading to a potential central serotonin deficiency that may underpin the mood disorders (anxiety and depression) after acute exposure and possibly also during ‘long COVID’. Increased glutamatergic activity by QA can lead to the cognitive dysfunction and neurological disorders associated with the virus.

The above account suggests that tryptophan metabolism via the KP appears to be the top pathway influenced by COVID-19 infection [61,106]. The demonstration in the many studies listed in Table 2 of changes in the [Kyn]/[Trp] ratio and other aspects of KP activation is further confirmed in the recently reported systematic review and meta-analytical study by Almulla et al [149]. Parameters including the [Kyn]/[Trp] ratio, [KA], and modulators of Trp disposition (albumin and NEFA) appear to determine various pathologies, severity, the need for intensive care, and death. Whereas depletion of NAD^+^ and ATP are the end result of COVID-19 infection, the elevation of kynurenic acid (KA) is an especially important earlier event, even-though this Kyn metabolite possesses both anti- and proinflammatory properties. The current status of the KP and related processes in COVID-19 based on available evidence is outlined in Figure 4, which is an updated version from an earlier study [34] and from which a number of therapeutic options could be explored with caution. These are (1) lowering plasma free [Trp] by albumin infusion and/or inhibitors of lipolysis, (2) prevention of proinflammatory cytokine elevation with antiinflammatory agents, (2) use of IDO inhibitors, (3) neutralisation of potential TDO induction by use of glucocorticoid antagonists, (4) prevention of the rise in KA by KAT inhibition, (5) prevention of 3-HAA and QA formation by KMO inhibition, (6) inhibition of IL-4I1, AhR and PARPs, (6) blocking upregulation of the salvage pathway, and (7) replenishing NAD^+^ with suitable precursors.

In hospitalised patients, a significant decrease in plasma albumin of ≥ 20% or a lesser decrease in association with elevated [NEFA] resulting in free Trp elevation would justify albumin infusion with or without antilipolytic therapy. The value of nicotinic acid as antilipolytic agent will be limited to the desired lowering free Trp levels, but will not extend to replenishing NAD^+^, as COVID-19 suppresses activity of the Preiss-Handler pathway [113]. Use of antiinflammatory agents that are known to suppress the cytokine elevation and/or of IDO1 inhibitors should be considered. In patients with raised cortisol levels, glucocorticoid antagonist therapy, already used clinically in other conditions, would be justified. Elevation of KA will require the use of KAT inhibitors. Many inhibitors have been developed, but their toxicity and cross reactivity towards other pyridoxal 5′-phosphate-dependent enzymes preclude their use in humans [150]. The active constituents of liquorice, glycyrrhizic acid, glycyrrhetinic acid and carbenoxolone are strong inhibitors of KAT II, acting by competing with the Kyn substrate [151]. Although identified for KAT II inhibition with a view to treating schizophrenia, which is associated with glutamatergic hypoactivity, these liquorice constituents should also be effective inhibitors of KAT II in peripheral tissues, where it is widely distributed. These liquorice constituents possess antiinflammatory and antiviral properties and inhibit liver fibrosis and tumor growth in cell cultures and experimental models (see [151] and references cited therein).

Currently, there are no IL-4I1 inhibitors suitable for use in humans, hence the need to develop suitable agents [29]. Similarly, strong AhR inhibitors are used experimentally [152], but a first-in-humans dose finding study of an AhR inhibitor (BAY2416964) in patients with advanced cancer is in progress [153]. Many PARP inhibitors have been developed and some have been approved for clinical use in cancer therapy [154]. The use of PARP inhibitors in COVID-19 therapy has been suggested by various investigators (see [34] and references cited therein). PARP inhibition will additionally prevent NAD^+^ depletion, thereby limiting SIRT inactivation. Blocking the up-regulation of the salvage pathway could be achieved by inhibition of NAMPT and NAMRK1-2. Finally, replenishing NAD^+^ levels can be achieved by using suitable NAD^+^ precursors. Careful choice of precursors is however important. Use of Trp is inadvisable for two reasons: increased production of Kyn metabolites and the powerful AhR agonist IPA. Similarly, nicotinic acid is unlikely to contribute to NAD^+^ replenishment, given that the Preiss-Handler pathway will also be blocked. This leaves nicotinamide or its riboside. Use of nicotinamide has been suggested by this and other authors, based on its antiinflammatory properties [34]. Although nicotinamide is a relatively weak PARP inhibitor, the increased production of NADP^+^ from the newly synthesised NAD^+^ provides a stronger PARP1 inhibitor. Whereas nicotinamide riboside (NAMR) is equally effective in increasing levels of NAD^+^(P^+^) as nicotinamide, it is 3-fold more active in increasing the ADP-ribose product of the PARP and SIRT reactions [155], an undesirable effect as PARPs are already activated by COVID-19. Also, NAMR lowers some, but not other, proinflammatory cytokine and chemokines [156]. A clinical trial of NAMR in elderly COVID-19 patients in Denmark (NCT04407390) was suspended due to recruitment issues. A completed trial involving the use of NAMR together with 3 nutritional substances: carnitine, serine and N-acetylcysteine [157] demonstrated rapid recovery, but, whereas elevation of some proinflammatory cytokines and chemokines was inhibited, assigning a particular effect to NAMR is difficult.

## Relevant studies with vaccines

An important question that has been minimally addressed is to what extent vaccine therapy is associated with inflammatory responses to COVID-19 that could impact Trp metabolism and whether interaction between vaccines and immune mediators is of diagnostic or prognostic value. In line with the assumed stimulation of the immune system by vaccines, IL-1β in human immune cells is raised by RNA vaccines by the RNA and the lipid formulation [158]. As stated above, IL-1β up-regulates KMO: the gateway for production of QA. A comparison of the Oxford/AstraZeneca [ChAdOx1] (AZ) and the Johnson & Johnson [Ad26.CoV2.S] COVID-19 vaccines [159] showed that the AZ vaccination increased several inflammatory and platelet activation markers more strongly than the mRNA vaccination, with the former preferentially increasing TNF-α, IL-1β and IL-8 and the latter vaccination increasing IL-6. CRP and IL-10 levels did not differ between groups. Various vaccine types at > single-dose levels have been shown to increase the above and other inflammatory cytokines (see [160] and references cited therein). Under vaccine conditions, Trp metabolism via IDO induction and initiated by proinflammatory cytokines can be expected and should be investigated. Whether incidence of complications in vaccine-receiving susceptible patients involves excessive immune activation causing augmented production of Kyn metabolites requires assessment that could reveal important diagnostic markers of disease severity. Combining vaccine therapy with biochemical investigations of Trp metabolism may widen our understanding of the immune effects of the vaccination strategy and provide useful indicators of diagnostic and prognostic values that could potentially enhance COVID-19 immunotherapy.

## Conclusions and comments

While most COVID-19 therapeutic efforts have understandably focused on vaccines from the public health perspective, less emphasis was directed at other therapeutic options either independently or conjointly with vaccination. Current evidence strongly suggests that the KP of Trp metabolism is the top target of COVID-19 infection and a major determinant of its pathophysiology. It is hoped that the present account has provided a stimulus to explore further the role of the KP in COVID-19 pathophysiology and immunotherapy. A number of therapeutic options require appraisal. The potential effects of antiinflammatory drugs on changes in the KP in COVID-19 are little explored. KAT inhibition would be justified, as the KA product initiates the AhR activation and the subsequent PARP activation. PARP inhibitor therapy could be equally important. The potential effects of vaccine therapy on the KP should be explored and may provide a new platform for future vaccine research.

## Abbreviations

CCL: chemokine C–C motif ligand
CSF: colony-stimulating factor
CTACK: cutaneous T-cell-attracting chemokine
CXCL: chemokine C-X-C motif ligand
GM-CSF: granulocyte colony stimulating factor
HGF: hepatocyte growth factor
IDO: indoleamine 2,3-dioxygenase
IFN: interferon
IL: interleukin
KA: kynurenic acid
KP: kynurenine pathway
Kyn: kynurenine
LIF: leukemia inhibitory factor
MCP: monocyte chemoattractant protein
M-CSF: macrophage colony stimulating factor
MIF: macrophage migration inhibitory factor
MIG: monokine-induced by interferon-γ
MIP: macrophage inflammatory protein
PARP: poly (ADP-ribose) polymerase
SCGF-β: stem cell growth factor β
SDF-1α: stromal cell-derived factor 1α
TDO: Trp 2,3-dioxygenase
TNF: tumour necrosis factor
Trp: tryptophan
